# Electronic cigarette use is negatively associated with body mass index: An observational study of electronic medical records

**DOI:** 10.1002/osp4.468

**Published:** 2020-12-22

**Authors:** Mohammed M. Alqahtani, Abdullah M. M. Alanazi, Abdulaziz S. Almutairi, Gregory Pavela

**Affiliations:** ^1^ King Saud Abdul‐Aziz University for Health Sciences Riyadh Saudi Arabia; ^2^ Department of Rehabilitation Science School of Health Profession University of Alabama at Birmingham Birmingham Alabama USA; ^3^ Immunology Department School of Biomedical Science University of Alabama at Birmingham Birmingham Alabama USA; ^4^ Department of Health Behavior School of Public Health University of Alabama at Birmingham Birmingham Alabama USA

**Keywords:** BMI, e‐cigarette use, patients, smoking, weight

## Abstract

**Objective:**

Vaping is advertised as a method to mitigate weight gain after smoking cessation; however, while there is an established inverse association between conventional tobacco use and body mass index (BMI), there is little research on the relationship between e‐cigarettes and BMI. This research tested whether e‐cigarette use was associated with BMI.

**Methods:**

A secondary data analysis of 207,117 electronic medical records from the UAB was conducted. Patient data from 1 September 2017 through 1 June 2018 were extracted. To be included in the analysis, a patient's record had to include measures of e‐cigarette use and key sociodemographic information. Ordinary least squares regression was used to test the association between e‐cigarette use and BMI, controlling for covariates; unconditional quantile regression was used to determine whether the association varied by BMI quantile. For comparison with tobacco smoking, the association between current tobacco smoking and BMI was estimated in a sample from the same population.

**Results:**

Respondents in the sample had an average BMI of 30.8 and average age of 50.0 years when BMI was measured. The sample was 51% female, 49.7% white, 46.7% black, and 1.0% Hispanic; 16.4% of the sample had less than a college education and approximately 5% reported currently using e‐cigarettes. Individuals who reported using e‐cigarettes had, on average, a lower BMI compared to those who did not report currently using e‐cigarettes; results indicated that this association did not significantly vary by BMI quantile. Individuals who reported being current smokers had a lower BMI, on average.

**Conclusion:**

These findings suggest that using e‐cigarettes is associated with a lower BMI in a population of individuals seeking health care, consistent with the association between conventional tobacco use and BMI. This study is a springboard for future research investigating the associations between e‐cigarette use, BMI, and risk of obesity in the general population.

## 1 INTRODUCTION

Conventional tobacco use is inversely associated with body mass index (BMI).^1^, ^2^ Whether e‐cigarette use is inversely associated with BMI is less certain.^3^ Like traditional cigarettes, e‐cigarettes provide substantial nicotine concentrations to users, ranging from 0 to 36 mg/mL.^4^ Nicotine may affect human body weight via several mechanisms. First, a growing body of literature indicates that nicotine obtained from smoking may suppress appetite.^5^ Following inhalation, nicotine reaches the brain in roughly 7 s.^6^ There, nicotine leads to the release of dopamine, norepinephrine, and serotine, neurotransmitters associated with appetite and pleasure.^7^ Nicotine may also influence body weight by increasing resting metabolic rate by 7% to 15%^8^ Additionally, nicotine elevates lipolysis and the subsequent recycling of fatty acids into triglycerides, which may ultimately lead to increased thermogenesis in adipose tissue.^9^

**FIGURE 1:**
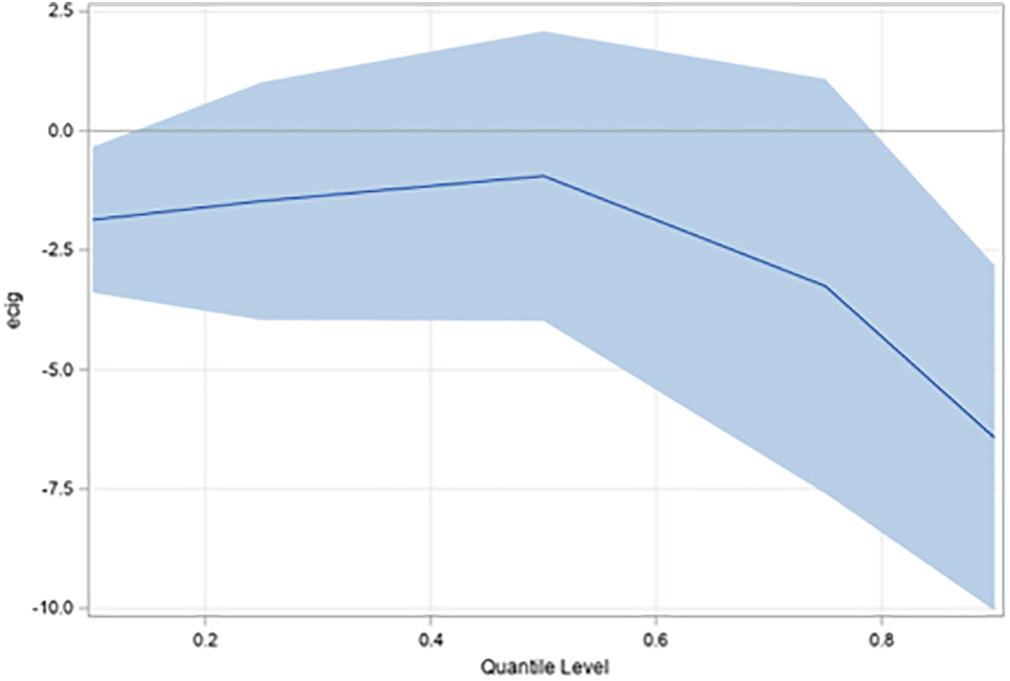
Estimated regression coefficients for e‐cig use predicting body mass index by quantile level for body mass index. Shaded areas represent 95% confidence intervals

Tobacco use is the leading cause of preventable death in the United States.^10^ However, electronic cigarettes, or “e‐cigarettes,” have emerged as a popular substitute for traditional tobacco products.^11^ First introduced to the US market in 2006, e‐cigarettes are available in an array of flavors.^11^, ^12^ Since their introduction, e‐cigarettes have gained popularity in all age groups, especially among teenagers. One study found that 20.8% (3.05 million) of high school students and 4.9% of middle school students (570,000) reported that e‐cigarettes were the most common tobacco product used by students of their age groups.^10^ E‐cigarette use is increasingly common in adults and especially prevalent among those who had never smoked cigarettes.^13^, ^14^ E‐cigarettes have grown in popularity for several reasons, including their use as aids smoking cessation, greater flexibility of public‐use, and the belief by many that they are a safer alternative to conventional tobacco products.^15^ Finally, the rapid uptake of e‐cigarettes has been facilitated by the relative lack of restrictions with regard to marketing and promotion compared to conventional cigarette products; in particular, e‐cigarette makers are currently allowed to show advertisements on television and online without restriction.^16^ Despite the increasing popularity of e‐cigarettes, there is limited knowledge regarding their metabolic effects.^3^ Further, tobacco smoking is the most common type of smoking globally,^17^ and e‐cigarette use is no exception, prompting the General Surgeon of the United States to issue a report about the risks of e‐cigarette use among adolescents.^18^ Massive public health campaigns have addressed the need for smoking cessation and proposed pharmacological and behavioral interventions that help smokers to quit. However, an enduring concern of smokers is weight gain after abstaining from cigarettes,^19^ especially among females.^20^ Thus, some studies have reported e‐cigarettes may serve as a smoking cessation aid.^21^, ^22^ For instance, one study found that the smoking cessation rate was 18.0% in those who tried e‐cigarettes, compared to 9.9% in the nicotine‐replacement group.^3^ However, not all studies have demonstrated e‐cigarettes to be an effective aid to smoking cessation,^22^ and it is not yet known whether e‐cigarettes share all the harmful effects of tobacco smoking on health, including insulin resistance, cytokine release, and an increased risk of metabolic syndrome, ultimately leading to cardiovascular complications.^3^ Recent evidence suggests that e‐cigarettes contain products harmful to human health, including formaldehyde, acetaldehyde, acrolein, propanol, acetone, and butanal.^23^ Some of these chemicals are produced when certain components of e‐cigarette liquids, like propylene glycerol and glycerin, are heated.^24^ Like traditional cigarettes, e‐cigarettes have long‐term cardiovascular and pulmonary effects, including chronic pulmonary inflammation, mucus hypersecretions, neutrophil inflammation, host defense reduction, and protease‐mediated lung tissue damage.^15^ Recent studies have also demonstrated that e‐cigarettes exacerbate airway physiology and the respiratory symptoms of pulmonary patients with asthma and chronic obstructive pulmonary disease (COPD). Indeed, inhalation of acrolein contributes to the development of COPD—that is, e‐cigarette use can cause throat irritation, coughing, and increased airway resistance.^14^, ^15^, ^24^ In brief, e‐cigarette users, in addition to nicotine, expose themselves to high levels of ultrafine particles and toxins that may increase the likelihood of cardiopulmonary diseases.^15^

Given the established inverse association between conventional tobacco use and BMI,^1^ and the likely effects of nicotine on weight in both conventional and e‐cigarettes, the purpose of this study is to test whether e‐cigarette use is negatively associated with BMI. While previous research has examined this association, finding that a higher BMI was associated with e‐cigarette and tobacco use, this research was limited to adolescents.^25^ Given the known inverse association between conventional tobacco use and BMI, the purpose of this study was to determine whether e‐cigarette use was associated with BMI, hypothesizing that e‐cigarette is negatively associated with BMI.

## 2 MATERIALS AND METHODS

Secondary data analysis was conducted using a dataset derived from 207,117 electronic medical records from the University of Alabama at Birmingham Informatics for Integrating Biology and the Bedside (i2b2). Patient data from 1 September 2017, through 1 June 2018, were extracted. i2b2 is an NIH‐funded National Center for Biomedical Computing based at Partners HealthCare System. i2b2 was developed as a scalable informatics framework designed for translational research. i2b2 was designed primarily for cohort identification, allowing users to perform an enterprise‐wide search on a de‐identified repository of health information to determine the existence of a set of patients meeting certain inclusion or exclusion criteria. i2b2 is a self‐service tool that enables researchers to access electronic health record data in the Cerner system significantly faster than requesting data from Enterprise Data Warehouse analysts.^26^ To be included in the analysis, a patient's record had to include measures of e‐cigarette use and key sociodemographic covariates: age at which BMI was assessed, sex, race/ethnicity, and educational attainment. Individuals with a BMI of less than 18.5 or greater than 100 were excluded from analyses. Ordinary least squares regression was used to test the association between e‐cigarette use and BMI, controlling for covariates. Further, to test whether the association between e‐cigarette use and BMI varies by BMI, unconditional quantile regression (UQR) was used to test whether the association varied at the 10th, 25%, 50%, 75th, and 90% BMI percentiles.^27^ To compare the association between e‐cigarette use and BMI with that of conventional tobacco smoking, the association between current tobacco smoking and BMI was estimated in a sample from the same population. Measures of conventional tobacco and e‐cigarette use were mutually exclusive; that is, participants with data on e‐cigarette use did not have data on conventional smoking use, thus, analyses examining the association between e‐cigarette and BMI and e‐cigarette use and BMI were run separately. The question used to measure e‐cigarette use was “Do you currently use electronic cigarettes or a vaping device?” Responses were dichotomized into “Yes” or “No.” A *p*‐value of ≤0.05 was considered statistically significant. Analyses were conducted in SAS 9.4.

## 3 RESULTS

The sample used to assess the relationship between e‐cigarette use and BMI included 965 patients (Table 1). Respondents in the sample had an average BMI of 30.8 and average age of 50.0 years when BMI was measured. The sample was 51% female, 49.7% white, 46.7% black, and 1.0% Hispanic; 16.4% of the sample had less than a college education and approximately 5% reported currently using e‐cigarettes. The sample used to assess the relationship between conventional tobacco use and BMI included 12,673 patients. Results from the model regressing BMI on e‐cigarette use (Table 2) indicated that e‐cigarette use was associated with a lower BMI (*b* = −3.07, *p* = 0.021). Results from the UQR (Table 3) suggested a U‐shaped relationship in the strength of the relationship between e‐cigarette use and BMI, such that the negative association was more consistently observed at lower and upper BMI quantiles; however, variation in the association between e‐cigarette use and BMI across BMI quantiles was not statistically significant (*χ* ^2^ = 8.07, df = 4, *p* = 0.0891) (Figure 1). Results from the model regressing BMI on conventional tobacco use (current smoker vs. otherwise), indicated that being a current smoker was associated with a lower BMI (*b* = −2.21, *p* = 0.021) (Table 4). Thus, both e‐cigarette and conventional tobacco use were inversely and significantly associated with BMI, after controlling for age, sex, race/ethnicity, and educational attainment.

**TABLE 1:** Basic characteristics of the population analyzed

Variable	*N*	Mean (SD)	Minimum	Maximum
E‐cigarette (0 no, 1 yes)	965	0.05 (0.21)	0	1
Female	965	0.51 (0.5)	0	1
White	965	0.50 (0.50)	0	1
Black	965	0.47 (0.50)	0	1
Hispanic	965	0.01 (0.10)	0	1
Other (refused)	965	0.02 (0.16)	0	1
Less than high school	965	0.16 (0.37)	0	1
High school degree	965	0.37 (0.48)	0	1
More than high school	965	0.25 (0.43)	0	1
College	965	0.21 (0.40)	0	1
Age (year)	965	49.95 (18.22)	13.00	93.00
BMI kg/m^2^	965	30.80 (8.60)	18.50	97.90

Abbreviation: BMI, body mass index.

**TABLE 2:** Results from an OLS model regressing BMI on E‐cigarette use

Variable	*B*	SEB	*t*	*p*
E‐cigarette use	−3.07	1.33	−2.31	0.0213
Female	2.48	0.56	4.42	<0.0001
Black	1.43	0.58	2.48	0.0131
Hispanic	−2.10	2.74	−0.77	0.4437
Other (refused)	−1.75	1.78	−0.98	0.3266
Less than high school	−1.77	0.93	−1.90	0.0577
High school	0.27	0.76	0.35	0.7270
More than high school	2.00	0.81	2.46	0.0141
Age	−0.04	0.016	−2.29	0.0221
Intercept	30.55	1.17	26.15	<0.0001

*Note: N* = 965. Adjusted *R* ^2^ = 0.0550.

Abbreviations: BMI, body mass index; OLS, ordinary least squares.

**TABLE 3:** Estimated regression coefficients for E‐cig use predicting body mass index by quantile level for body mass (sample size: 965)^a^

	*t* = 0.10		*t* = 0.25		*t* = 0.50		*t* = 0.75		*t* = 0.90	
	*B*	SE, *p*	*B*	SE, *p*	*B*	SE, *p*	*B*	SE, *p*	*B*	SE, *p*
Intercept	21.6	1.1, <0.0001	24.2	1.9, <0.0001	28.6	1.1, <0.0001	32.4	1.9, <0.0001	41.3	2.6, <0.0001
E‐cigarette	−1.9	0.8, 0.017	−1.5	1.3, 0.248	−0.9	1.5, 0.542	−3.3	2.2, 0.142	−6.4	1.8, <0.0001

Abbreviation: BMI, body mass index.

^a^ The association between e‐cigarette use and BMI did not significantly vary across BMI quantiles (*χ* ^2^ = 8.07, df = 4, *p* = 0.0891).

**TABLE 4:** Results from an OLS model regressing BMI on tobacco use

Variable	*B*	SEB	*t*	*p*
Current smokers	−2.22	0.16	13.56	<0.0001
Female	2.09	0.13	15.63	<0.0001
Black	1.74	0.14	12.36	<0.0001
Hispanic	−1.31	0.53	−2.47	0.0134
Other/refused	−1.33	0.38	−3.55	0.0004
Less than high school	−0.19	0.24	−0.82	0.4124
High school	0.28	0.18	1.60	0.1103
More than high school	0.53	0.18	2.86	0.0043
Age	−0.01	0.0003	−2.38	0.0175
Intercept	29.12	0.29	101.99	<0.0001

*Note: N* = 12,674. Adjusted *R* ^2^ = 0.0573

Abbreviations: BMI, body mass index; OLS, ordinary least squares.

## 4 DISCUSSION

This study demonstrated that e‐cigarette use is associated with lower BMI among adults using data derived from electronic medical records. This study also found that current conventional cigarette use—a positive control given its known association with BMI—was inversely associated with BMI. This suggests—but does not demonstrate—a shared mechanism through which both forms of smoking are associated with a lower BMI, either causally (e.g., via the effects of nicotine on body weight), or spuriously (e.g., thinner individuals are more likely to smoke). On the one hand, both conventional tobacco smoking and e‐cigarettes may affect weight through the effects of nicotine.^5^ On the other hand, tobacco cigarettes and e‐cigarette deliver nicotine in differing concentrations and alongside a differing set of chemicals, potentially leading to the same effect on weight but through different mechanisms. In particular, different additive flavors in the nicotine solution of e‐cigarettes may exert different effects on appetite.^5^, ^29^ ^28^ Future research should further explore these differences, including nicotine concentration and flavor addition, with respect to their effects on BMI in e‐cigarette users.

Despite the many health concerns associated with both conventional tobacco use and e‐cigarettes, marketing efforts have leveraged the common perception that smoking is associated with lower body weight, as data from several sources indicate an increasing number of advertisements that link tobacco use with lower weight.^5^ This marketing strategy has made vaping more attractive to those who may have concerns about their weight, for instance, a recent study has yielded that individuals who reported vaping for weight loss/control (13.5%) were more likely to vape frequently, be overweight; and restrict calories.^29^ Several studies have linked weight concerns with increased e‐cigarette use. For instance, a recent study showed weight concern was associated with greater e‐cigarette use among adults,^30^ despite the many deleterious effects of nicotine. The uptake of e‐cigarette use in recent years raises questions about whether e‐cigarette use is a less harmful option for those who want to quit cigarette smoking.^21^, ^32^ ^31^ This concern also involves whether e‐cigarette use is gateway for people seeking to control their weight.^19^, ^33^ ^32^ Given this interest, it is essential to emphasize that nicotine has many deleterious effects on health in addition to its possible effects on weight. High nicotine concentrations can cause acute toxicity, increasing the risk of disease by activating multiple biological pathways.^9^ Nicotine has been linked to different cancers, specifically oral, esophageal, and pancreatic cancer.^33^ The adverse effects of nicotine are also observed in the developing fetus, affecting brain development and increasing the risk of pre‐term delivery or stillbirths.^9^ Thus, results from this study should not be interpreted as warranting the use of e‐cigarettes for its potential effects on weight, given both the limitations of out observational methodology and the known harmful effects of e‐cigarettes generally and nicotine specifically.

The population studied in this research is made up of individuals receiving health care; as a result, the generalizability of the findings is limited. Because i2b2 data are cross‐sectional, the temporality of association between e‐cigarette use and BMI cannot be inferred. Further, whether those who use e‐cigarettes in the analyses were former smokers or dual users of tobacco cigarette and e‐cigarettes use cannot be determined. Moreover, whether weight‐related concerns influenced their decision to use e‐cigarettes was not determined. Future research would benefit from a more representative sample with measures of both conventional tobacco use and e‐cigarette to better isolate the association between e‐cigarette use and BMI in the general population and estimate the association between dual use of e‐cigarettes and conventional tobacco on BMI.

## 5 CONCLUSION

There is little research on the relationship between e‐cigarette use and BMI. These findings suggest that using e‐cigarette is associated with lower BMI in a population of individuals seeking health care, after controlling for age, race/ethnicity, sex, and education.

## CONFLICT OF INTEREST

The authors declared no conflict of interest.

## AUTHOR CONTRIBUTIONS

Mohammed M. Alqahtani did the literature review, data collection, study design, analysis of data, and manuscript preparation and writing. Abdullah M. M. Alanazi and Abdulaziz S. Almutairi contributed to the literature search, and manuscript preparation and editing. Gregory Pavela assisted with the study design, writing the manuscript, and data analysis and mentorship to the student author.
